# Perioperative outcomes of esophagectomy after doublet versus docetaxel‐based triplet neoadjuvant chemotherapy in older patients: A nationwide inpatient database study in Japan

**DOI:** 10.1002/ags3.70000

**Published:** 2025-02-05

**Authors:** Yuki Hirano, Takaaki Konishi, Hidehiro Kaneko, Satoru Matsuda, Hirofumi Kawakubo, Yuya Kimura, Hiroki Matsui, Kiyohide Fushimi, Hiroyuki Daiko, Osamu Itano, Hideo Yasunaga, Yuko Kitagawa

**Affiliations:** ^1^ Department of Hepatobiliary–Pancreatic and Gastrointestinal Surgery International University of Health and Welfare School of Medicine Chiba Japan; ^2^ Department of Clinical Epidemiology and Health Economics School of Public Health, the University of Tokyo Tokyo Japan; ^3^ Department of Cardiovascular Medicine The University of Tokyo Tokyo Japan; ^4^ Department of Surgery Keio University School of Medicine Tokyo Japan; ^5^ Department of Health Policy and Informatics Tokyo Medical and Dental University Graduate School Tokyo Japan; ^6^ Division of Esophageal Surgery National Cancer Center Hospital Tokyo Japan

**Keywords:** doublet, esophageal cancer, esophagectomy, neoadjuvant chemotherapy, older patients, triplet

## Abstract

**Background:**

Although docetaxel‐based triplet neoadjuvant chemotherapy has yielded promising results for locally advanced esophageal cancer, there are concerns that the triplet regimen can increase perioperative adverse events in older patients. This retrospective study assessed the perioperative outcomes following doublet or docetaxel‐based triplet chemotherapy and esophagectomy in older patients.

**Methods:**

The data of patients aged 70–79 years who received cisplatin and 5‐fluorouracil (CF) or docetaxel, cisplatin, and 5‐fluorouracil (DCF) before esophagectomy were extracted from a nationwide Japanese inpatient database (April 2012–March 2022). The primary outcomes were major and respiratory complications. The secondary outcomes included anastomotic leakage, 30‐day unplanned readmission, and 30‐ and 90‐day mortality. Analyses were conducted using overlap propensity score weighting, propensity score matching, and instrumental variable methods to adjust for potential confounders.

**Results:**

Of 5229 eligible patients, 3457 (66%) and 1772 (34%) patients received neoadjuvant CF and DCF, respectively. Major and respiratory complications occurred in 5229 (40%) and 1388 (27%) patients, respectively. After overlap weighting, DCF was not associated with a higher frequency of major (odds ratio 0.99 [95% confidence interval 0.87–1.12]) and respiratory complications (odds ratio 1.04 [0.90–1.19]) compared with CF. The frequencies of anastomotic leakage, 30‐day unplanned readmission, and 30‐ and 90‐day mortality did not differ between the groups. Propensity score matching and instrumental variable analyses yielded similar results.

**Conclusions:**

Neoadjuvant DCF was not associated with a higher frequency of perioperative adverse events compared with CF after esophagectomy in patients aged 70–79 years.

## Graphical abstract

In this nationwide inpatient database study of 5229 patients aged 70–79 years who underwent esophagectomy for cancer after neoadjuvant chemotherapy, overlap propensity score weighting analysis showed that neoadjuvant docetaxel‐based triplet regimen (docetaxel, cisplatin, and 5‐fluorouracil) was not associated with a higher incidence of perioperative adverse events (major and respiratory complications, mortality, and 30‐day unplanned readmission) compared with the doublet regimen (cisplatin and 5‐fluorouracil). Propensity score matching and instrumental variable analyses yielded similar results.

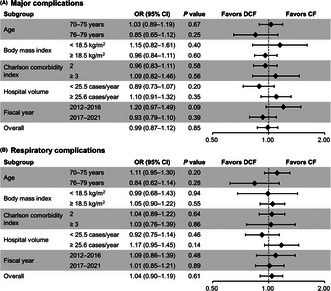

## 1 INTRODUCTION

Neoadjuvant therapy followed by esophagectomy constitutes the standard care for locally advanced esophageal cancer worldwide. In Western countries, chemoradiotherapy has been adopted as a standard neoadjuvant therapy^1^ and neoadjuvant chemotherapy (NAC) is considered an alternative option.^2^, ^3^ A recent European multi‐center observational study enrolling 1032 patients aged ≥65 years demonstrated that NAC resulted in lower postoperative complications and improved overall survival compared with neoadjuvant chemoradiotherapy.^2^ Among the NAC protocols, a docetaxel‐based triplet regimen (docetaxel, oxaliplatin, and 5‐fluorouracil/leucovorin [FLOT]) has shown promising results since 2017.^3^ Recently, the ESOPEC trial revealed that perioperative FLOT showed better overall survival than neoadjuvant chemoradiotherapy.^4^ While esophageal adenocarcinoma is common in Western countries, squamous cell carcinoma is dominant among the East Asian population.^5^ In Japan, NAC with a doublet regimen (cisplatin and 5‐fluorouracil [CF]) has been the standard treatment since the JCOG9907 trial (neoadjuvant vs. adjuvant CF).^6^ A docetaxel‐based triplet regimen (docetaxel, cisplatin, and 5‐fluorouracil [DCF]) has become the new standard since 2022,^5^ based on the JCOG 1109 NExT trial's results (neoadjuvant CF vs. DCF vs. CF plus radiotherapy).^7^

Although docetaxel‐based triplet NAC has shown promising results for locally advanced esophageal cancer,^3^, ^4^, ^7^, ^8^ its administration in older patients is controversial owing to the lack of evidence. Previous randomized controlled trials of docetaxel‐based triplet NAC in patients with esophageal cancer included a limited proportion of patients aged above 70 years, especially those older than 75 years.^3^, ^7^, ^9^ A recent retrospective study encompassing 85 hospitals accredited by the Japan Esophageal Society reported that DCF was associated with a better pathological response compared with CF, but may not contribute to better overall survival due to increased postoperative complications, especially pneumonia, in patients aged >75 years (*n* = 156 in 2010–2015).^10^ However, this study was limited by the failure to adjust for comorbidities and activities of daily living as background factors. The International Society of Geriatric Oncology emphasized the importance of including these factors in treatment decision‐making for older patients with cancer, due to the heterogeneity of the aging process.^11^ Additionally, because the management of chemotherapy‐related toxicities has improved over the past decade (e.g., prophylactic administration of granulocyte colony‐stimulating factor, development of long‐acting granulocyte colony‐stimulating factor, and adjustments of chemotherapy dosage), the deleterious effect of DCF may have decreased.

Therefore, this study aimed to compare the perioperative outcomes of esophagectomy in older patients who received neoadjuvant CF or DCF, using the latest nationwide inpatient database from Japan.

## 2 METHODS

### 2.1 Database

This retrospective cohort study extracted data from the Diagnosis Procedure Combination database, a Japanese nationwide repository of inpatient information.^12^ This database collects more than 8 million discharge summaries and hospital administrative claims data annually from more than 1200 hospitals. Participation in the database is mandatory for all universities and voluntary for other hospitals.

The Diagnosis Procedure Combination database contains information on the following variables: sex; age; body mass index; smoking index; diagnosis and comorbidities on admission and complications after admission, which were recorded using the International Classification of Diseases, Tenth Revision (ICD‐10) codes; clinical cancer stage according to the seventh edition of the Union for International Cancer Control Tumor, Node, Metastasis classification; use of preoperative chemotherapy/radiotherapy; medications administered during hospitalization; interventional/surgical procedures recorded using the original Japanese codes; unique hospital identifier; discharge status; and hospitalization costs. Each patient's discharge summary is coded by the attending physicians. Previous validation studies have shown that this database contains highly accurate data on patients with esophageal cancer,^13^ surgical procedures including oncologic esophagectomy,^14^ comorbidities,^15^ and postoperative complications.^16^

### 2.2 Study protocol

The data of patients who underwent esophagectomy for esophageal cancer were extracted from the database (April 2012–March 2022). Esophagectomy with two‐field (thoraco‐abdominal) or three‐field (cervico‐thoraco‐abdominal) lymph node dissection was identified using the original Japanese procedure codes, with the exception of transhiatal esophagectomy and two‐stage reconstruction.^17^, ^18^, ^19^, ^20^, ^21^ We included patients who were aged 70–79 years at admission for initial NAC and commenced CF or DCF 3–20 weeks before esophagectomy. We did not include patients aged >80 years because they rarely received neoadjuvant DCF during the study period and may not be comparable with those receiving neoadjuvant CF. The exclusion criteria were as follows: missing data on body mass index; presence of chronic kidney disease; radiotherapy performed within 1 year before esophagectomy; use of anticancer drugs other than docetaxel, cisplatin, or 5‐fluorouracil within 20 weeks before esophagectomy; split‐dose (daily/weekly/biweekly) cisplatin administration (because the standard regimen for esophageal cancer was three‐weekly in Japan^6^, ^7^); concomitant surgery for laryngeal or hypopharyngeal cancer; intestinal reconstruction; and performance of surgery at hospitals that performed less than five esophagectomies per year during the study period. Patients with supraclavicular lymph node metastasis (M1/stage IV disease) were included, as this is considered curable in Japan.^5^

The study compared patients who received neoadjuvant CF (CF group) and those who received neoadjuvant DCF (DCF group). We assessed the cycles of NAC (defined as the frequency of cisplatin administration).

The primary outcomes were major postoperative complications (comprising respiratory complications, anastomotic leakage, pneumothorax, chylothorax, empyema, peritonitis, ileus, bowel obstruction, symptomatic hiatal or diaphragmatic hernia, pulmonary embolism, acute coronary syndrome, heart failure, stroke, acute kidney injury, sepsis, and others resulting in reintubation or death)^17^, ^18^, ^19^, ^20^ and respiratory complications. The secondary outcomes included respiratory failure (defined as mechanical ventilation use lasting >2 days following surgery),^18^, ^20^, ^21^ anastomotic leakage, postoperative length of stay, total hospitalization cost, 30‐day unplanned readmission (unplanned readmission within 30 days of discharge), 30‐day mortality (death within 30 days after surgery, including death before discharge and during readmission), and 90‐day mortality (death within 90 days after surgery, including deaths before discharge and during readmission). Patients discharged after death were excluded from the analyses of readmission. Additionally, adverse events during NAC, including febrile neutropenia and weight loss,^19^ and delayed surgery (defined as surgery performed more than 8 weeks after the last administration of chemotherapy)^7^ were assessed. Table S1 and Appendix S1 provide detailed definitions of the outcomes.

### 2.3 Statistical analysis

Overlap propensity score weighting analysis^22^ was used to compare the outcomes between the CF and DCF groups. This method adjusts for the measured potential confounding variables without excluding participants from the original sample. This method can avert some potential limitations of classic propensity scoring methods (e.g., inverse probability of treatment weighting and matching) with respect to the target population, covariate balance, and precision, and mimics the properties of a randomized clinical trial.^22^ The propensity score was estimated using a multivariable logistic regression model based on the patient‐related background factors prior to NAC (sex, age, body mass index,^17^ activities of daily living [Barthel index, inability to walk independently >45 m], pre‐NAC tube feeding,^19^ smoking index, Charlson comorbidity index, hypertension, diabetes mellitus, chronic obstructive pulmonary disease, liver disease, clinical T factor, clinical N factor, and clinical M factor) and hospital‐related factors (hospital type, hospital volume, hospitals' early extubation proportion,^20^ hospitals' minimally invasive esophagectomy proportion, and fiscal year). Detailed definitions of the background factors are presented in Appendix S2. To examine the balance in the baseline background factors between the two groups before and after adjustment, absolute standardized differences were calculated: a difference of less than 10% appropriate balance between the groups.^23^ We subjected the overlap‐weighted cohorts to univariable regression analyses: logistic regression analyses were performed for binary outcomes, and linear regression analyses for continuous outcomes.

We performed five sensitivity analyses to confirm the results of the primary outcomes of the main analysis. First, 1:1 propensity score matching was conducted to compare the outcomes between the two groups.^23^ We used a nearest neighbor matching algorithm within a caliper ≤0.2 of the pooled standard deviation of the estimated logits without replacement. Second, multivariable logistic regression analyses were performed. We fitted generalized estimating equations to adjust for within‐hospital clustering, such as the patients' characteristics or physicians' practice patterns within the same hospital.^24^ The explanatory variables were the same as those used for estimating the propensity score. Third, instrumental variable analyses were conducted to adjust for the unmeasured background factors.^25^ Although propensity score analysis and multivariable regression analysis cannot eliminate hidden biases introduced by unmeasured confounders, instrumental variable analysis can theoretically adjust for both measured and unmeasured confounders, thereby mimicking attributes of a randomized clinical trial.^25^ Since the “facility treatment rate” is the best‐known type of instrumental variable,^25^ we designated the “biennial rate of selecting neoadjuvant DCF among eligible patients in a facility” as the instrumental variable. In the instrumental variable analysis, facilities with less than 20 eligible cases during the study period were excluded. A two‐stage residual inclusion method was used, using the same explanatory variables as those in the main analysis. An F‐statistic >10 indicates that the instrument is valid.^25^ Fourth, we redefined the CF and DCF groups based on the reception of ≥2 cycles of NAC, followed by overlap weighting analysis. Finally, we performed an overlap weighting analysis, excluding patients who underwent delayed surgery.

Additionally, subgroup analyses using overlap weighting stratified by age (70–75 or 76–79 years), body mass index (<18.5 or ≥18.5 kg/m^2^), Charlson comorbidity index (2 or ≥3), hospital volume (stratified by the median hospital volume), and fiscal year (2012–2016 or 2017–2021) were performed. The subgroup‐specific propensity score was calculated for each subgroup analysis.

The *t*‐test was used to compare continuous variables, and the chi‐squared test for the comparison of categorical variables between the groups. The Cochran–Armitage trend test was used to evaluate the change in the proportion of patients receiving DCF over the study period. Statistical significance was set at *p* < 0.050. All statistical analyses were conducted using STATA version 17 (StataCorp LLC, College Station, TX, USA).

## 3 RESULTS

Overall, 47 781 patients who underwent oncological esophagectomy between April 2012 and March 2022 were identified. Of them, 7487 patients were aged 70–79 years and received neoadjuvant CF or DCF 3–20 weeks prior to esophagectomy. We excluded 2258 patients who met the following exclusion criteria: missing data on body mass index (*n* = 26), chronic kidney disease (*n* = 52), radiotherapy (*n* = 645), use of other anticancer drugs (*n* = 147), split‐dose cisplatin regimen (*n* = 337), combined surgery for laryngeal or hypopharyngeal cancer (*n* = 60), intestinal reconstruction (*n* = 157), and surgery at low‐volume hospitals (*n* = 834). Finally, we analyzed 5229 patients with a median age of 73 years across 265 hospitals.

The CF regimen was administered to 3457 (66%) patients across 249 hospitals, while 1772 (34%) patients across 157 hospitals received the DCF regimen. The proportion of patients who received one, two, and more than three cycles of NAC was 23%, 76%, and 1.2% in the CF group, and 15%, 55%, and 30% in the DCF group, respectively. During the study period, the number of older patients receiving NAC (either CF or DCF) increased from 627 in 2012–2013 to 1311 in 2020–2021. Figure S1 shows the trend of increasing DCF administration over time in older patients receiving NAC (22% in 2012 to 40% in 2021; *p* < 0.001).

Table 1 shows the background characteristics before and after overlap weighting. Before overlap weighting, DCF was more likely to be used in the following conditions compared with CF: patients aged 70–71 years; those on pre‐NAC tube feeding; Barthel index <95, inability to walk independently; clinical T3, T4, N2–3, and M1; treatment implemented at a non‐teaching hospital; treatment at a high‐volume hospital; and treatment period of 2020–2021. After overlap weighting, the background factors were well balanced between the groups.

**TABLE 1:** Patient‐ and hospital‐related characteristics before and after overlap weighting.

Variable	Before overlap weighting			After overlap weighting^a^		
	CF (*n* = 3457)	DCF (*n* = 1772)	ASD	CF (*n* = 2609)	DCF (*n* = 2609)	ASD
Sex, male	2882 (83)	1517 (86)	6.2	(85)	(85)	0.0
Age, years						
70–71	912 (26)	574 (32)	13	(30)	(30)	0.0
72–73	850 (25)	499 (28)	8.1	(28)	(28)	0.0
74–75	747 (22)	358 (20)	3.5	(21)	(21)	0.0
76–77	596 (17)	225 (13)	13	(14)	(14)	0.0
78–79	352 (10)	116 (6.5)	13	(7.8)	(7.8)	0.0
Body mass index, kg/m^2^						
<16.0	82 (2.4)	60 (3.4)	6.1	(3.0)	(3.0)	0.0
16.0–18.4	398 (12)	226 (13)	3.8	(12)	(12)	0.0
18.5–22.9	1781 (52)	955 (54)	4.8	(54)	(54)	0.0
23.0–27.4	1075 (31)	476 (27)	9.3	(28)	(28)	0.0
≥27.5	121 (3.5)	55 (3.1)	2.2	(3.3)	(3.3)	0.0
Activities of daily living						
Barthel index <95	84 (2.4)	124 (7.0)	22	(3.7)	(3.7)	0.0
Inability to walk independently (>45 m)	59 (1.7)	102 (5.8)	21	(2.9)	(2.9)	0.0
Pre‐NAC tube feeding	130 (3.8)	137 (7.7)	17	(5.6)	(5.6)	0.0
Smoking index, pack‐years						
0–5	1018 (29)	535 (30)	1.6	(29)	(29)	0.0
6–20	373 (11)	186 (10)	1.0	(11)	(11)	0.0
21–40	723 (21)	362 (20)	1.2	(21)	(21)	0.0
≥41	953 (28)	521 (29)	4.1	(29)	(29)	0.0
Missing	390 (11)	168 (9.5)	5.9	(10)	(10)	0.0
Comorbidities						
Charlson comorbidity index						
2	2753 (80)	1410 (80)	0.2	(80)	(80)	0.0
3–4	500 (14)	234 (13)	3.6	(13)	(13)	0.0
≥5	204 (5.9)	128 (7.2)	5.3	(6.9)	(6.9)	0.0
Hypertension	949 (27)	503 (28)	2.1	(28)	(28)	0.0
Diabetes mellitus	503 (15)	226 (13)	5.2	(13)	(13)	0.0
Chronic obstructive pulmonary disease	109 (3.2)	37 (2.1)	6.7	(2.3)	(2.3)	0.0
Liver disease	164 (4.7)	53 (3.0)	9.1	(3.5)	(3.5)	0.0
Clinical T factor						
T1	540 (16)	157 (8.9)	21	(12)	(12)	0.0
T2	781 (23)	255 (14)	21	(17)	(17)	0.0
T3	1827 (53)	1095 (62)	18	(59)	(59)	0.0
T4	111 (3.2)	154 (8.7)	23	(5.5)	(5.5)	0.0
TX/missing	198 (5.7)	111 (6.3)	2.3	(6.3)	(6.3)	0.0
Clinical N factor						
N0	955 (28)	378 (21)	15	(24)	(24)	0.0
N1	1428 (41)	667 (38)	7.5	(39)	(39)	0.0
N2–3	870 (25)	625 (35)	22	(32)	(32)	0.0
NX/missing	204 (5.9)	102 (5.8)	0.6	(6.1)	(6.1)	0.0
Clinical M factor						
M0	3144 (91)	1512 (85)	17	(88)	(88)	0.0
M1	112 (3.2)	148 (8.4)	22	(5.4)	(5.4)	0.0
MX/missing	201 (5.8)	112 (6.3)	2.1	(6.5)	(6.5)	0.0
Teaching hospital	1273 (37)	526 (30)	15	(34)	(34)	0.0
Hospital volume, cases/year						
5.0–11.0	822 (24)	219 (12)	30	(17)	(17)	0.0
11.1–19.4	703 (20)	319 (18)	5.9	(21)	(21)	0.0
19.5–31.5	681 (20)	370 (21)	2.9	(22)	(22)	0.0
31.6–73.0	694 (20)	349 (20)	1.0	(21)	(21)	0.0
≥73.1	557 (16)	515 (29)	31	(20)	(20)	0.0
Hospitals' early extubation proportion						
0%–9%	641 (19)	403 (23)	10	(23)	(23)	0.0
10%–20%	539 (16)	499 (28)	31	(20)	(20)	0.0
21%–72%	736 (21)	305 (17)	10	(20)	(20)	0.0
73%–94%	715 (21)	284 (16)	12	(19)	(19)	0.0
≥95%	826 (24)	281 (16)	20	(18)	(18)	0.0
Hospitals' MIE proportion						
0%–50%	656 (19)	344 (19)	1.1	(21)	(21)	0.0
51%–63%	622 (18)	454 (26)	19	(21)	(21)	0.0
64%–74%	685 (20)	361 (20)	1.4	(21)	(21)	0.0
75%–86%	640 (19)	401 (23)	10	(21)	(21)	0.0
≥87%	845 (24)	210 (12)	33	(16)	(16)	0.0
Fiscal year						
2012–2013	455 (13)	172 (9.7)	11	(11)	(11)	0.0
2014–2015	653 (19)	281 (16)	8.0	(17)	(17)	0.0
2016–2017	763 (22)	347 (20)	6.1	(20)	(20)	0.0
2018–2019	794 (23)	453 (26)	6.1	(25)	(25)	0.0
2020–2021	792 (23)	519 (29)	15	(28)	(28)	0.0

*Note*: Data are presented as *n* (%).

Abbreviations: ASD, absolute standardized difference; CF, cisplatin, 5‐fluorouracil; DCF, docetaxel, cisplatin, 5‐fluorouracil; MIE, minimally invasive esophagectomy; NAC, neoadjuvant chemotherapy.

^a^ After overlap weighting, one individual no longer represents one data entity and, thus, raw counts are not reported after overlap weighting.

Table 2 presents the primary and secondary outcomes. Major complications occurred in 2074 of 5229 (40%) patients: 1364 (39%) patients in the CF group and 713 (40%) in the DCF group. Respiratory complications occurred in 1388 (27%) patients: 893 (26%) patients in the CF group and 495 (28%) in the DCF group. After overlap weighting, the frequency of major and respiratory complications did not differ significantly between the CF and DCF groups: major complications, 41% versus 41% (odds ratio 0.99 [95% confidence interval 0.87–1.12]); and respiratory complications 27% vs. 27% (odds ratio 1.04 [0.90–1.19]). The proportions of respiratory failure, anastomotic leakage, 30‐day unplanned readmission, 30‐day mortality, 90‐day mortality, length of stay after surgery, and total hospitalization cost were also similar between the two groups.

**TABLE 2:** Perioperative outcomes before and after overlap weighting.

Outcome	Before overlap weighting		*p* Value	After overlap weighting^a^			*p* Value
	CF (*n* = 3457)	DCF (*n* = 1772)		CF (*n* = 2609)	DCF (*n* = 2609)	Odds ratio or coefficient (95% CI)	
Major complications	1361 (39)	713 (40)	0.54	(41)	(41)	0.99 (0.87 to 1.12)	0.85
Respiratory complications	893 (26)	495 (28)	0.10	(27)	(28)	1.04 (0.90 to 1.19)	0.61
Respiratory failure^b^	429 (12)	246 (14)	0.13	(12)	(14)	1.16 (0.96 to 1.39)	0.12
Anastomotic leakage	552 (16)	268 (15)	0.43	(16)	(15)	0.98 (0.82 to 1.17)	0.83
30‐day unplanned readmission^c^	253 (7.5)	121 (7.0)	0.51	(7.5)	(7.9)	1.06 (0.83 to 1.35)	0.65
30‐day mortality	21 (0.6)	8 (0.5)	0.47	(0.5)	(0.5)	1.08 (0.44 to 2.63)	0.86
90‐day mortality	57 (1.7)	24 (1.4)	0.41	(1.4)	(1.5)	1.04 (0.62 to 1.76)	0.87
Length of stay after surgery, days	24 (17 to 38)	24 (18 to 37)	0.87	24 (17 to 38)	24 (17 to 37)	0.7 (−1.6 to 2.9)	0.57
Total hospitalization cost, US$	21 246 (18 676 to 26 037)	21 995 (19 542 to 27 262)	0.034	21 589 (18 949 to 26 423)	21 698 (19 272 to 27 024)	278 (−558 to 1114)	0.51

*Note*: Data are presented as *n* (%) or median (interquartile range). Odds ratios or coefficients are calculated with reference to patients in the CF group.

Abbreviations: CF, cisplatin, 5‐fluorouracil; CI, confidence interval; DCF, docetaxel cisplatin, 5‐fluorouracil.

^a^ After overlap weighting, one individual no longer represents one data entity; thus, the raw counts are not reported after overlap weighting.

^b^ Respiratory failure was defined as mechanical ventilation use lasting >2 days following surgery.

^c^ To avoid immortal time bias, we excluded 91 patients who were discharged after death from the outcome analysis.

Table S2 shows the adverse events during NAC and delayed surgery. After overlap weighting, the frequency of febrile neutropenia was higher in the DCF group than in the CF group (1.1% vs. 17%; *p* < 0.001). The frequency of weight loss ≥5% during NAC was similar between the two groups (26% vs. 25%; *p* = 0.32), although the median weight loss during NAC was 1.1% lower in the DCF group than in the CF group (*p* < 0.001). The proportion of delayed surgery did not differ significantly between the CF and DCF groups (8.5% vs. 9.6%, *p* = 0.27).

The results of sensitivity analyses were similar to those of the main analysis (Table 3). Propensity score matching yielded 1425 pairs (Table S3 shows the background characteristics). The *F*‐statistic in the instrumental variable analysis was 880, indicating that the biennial rate of selecting neoadjuvant DCF was a valid instrumental variable. Tables S4 and S5 show the background characteristics before and after overlap weighting using the redefined model and excluding patients with delayed surgery, respectively.

**TABLE 3:** Sensitivity analyses for the primary outcomes.

	Major complications		Respiratory complications	
	Odds ratio (95% CI)	*p* Value	Odds ratio (95% CI)	*p* Value
Overlap weighting	0.99 (0.87–1.12)	0.85	1.04 (0.90–1.19)	0.61
Propensity score matching	1.00 (0.86–1.16)	1.00	1.06 (0.90–1.25)	0.48
Multivariable logistic regression^a^	0.95 (0.79–1.13)	0.54	0.95 (0.77–1.18)	0.64
Instrumental variable^b^	1.13 (0.80–1.60)	0.49	1.18 (0.76–1.85)	0.46
Overlap weighting using the redefined model^c^	1.01 (0.87–1.16)	0.93	1.08 (0.92–1.26)	0.36
Overlap weighting excluding patients who underwent delayed surgery^d^	0.97 (0.85–1.11)	0.68	1.00 (0.86–1.16)	0.97

*Note*: Odds ratios are calculated with reference to patients in the CF group.

Abbreviations: CF, cisplatin, 5‐fluorouracil; CI, confidence interval; DCF, docetaxel, cisplatin, 5‐fluorouracil.

^a^ The explanatory variables were sex, age, body mass index, Barthel index, inability to walk independently (>45 m), pre‐neoadjuvant chemotherapy tube feeding, smoking index, Charlson comorbidity index, hypertension, diabetes mellitus, chronic obstructive pulmonary disease, liver disease, clinical T factor, clinical N factor, clinical M factor, hospital type, hospital volume, hospitals' early extubation proportion, hospitals' minimally invasive esophagectomy proportion, and fiscal year. Generalized estimating equations were used to adjust for within‐hospital clustering.

^b^ The “biennial rate of selecting neoadjuvant DCF among eligible patients in a facility” was set as the instrumental variable. The explanatory variables were the same as those used in the multivariable regression, with the residuals calculated for each patient based on the difference between the model‐predicted probability of receiving DCF and the actual receipt of DCF. Generalized estimating equations were used to adjust for within‐hospital clustering.

^c^ The redefined model only included patients who received ≥2 cycles of neoadjuvant chemotherapy (*n* = 4172). The background characteristics are shown in Table S4.

^d^ We excluded 434 patients who underwent delayed surgery (defined as surgery performed more than 8 weeks after the last administration of chemotherapy). The background characteristics are presented in Table S5.

**FIGURE 1:**
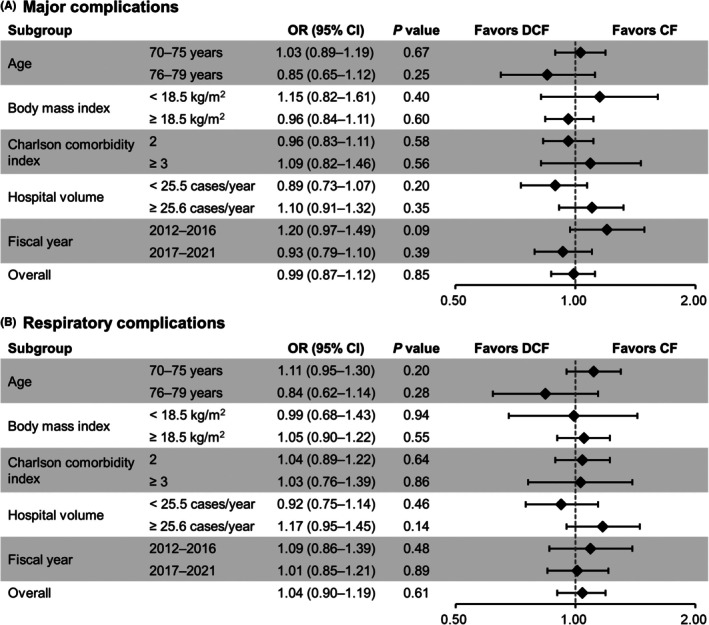
Subgroup analyses of primary outcomes in overlap weighted patients. Odds ratios (ORs) of major complications (A) and respiratory complications (B) associated with docetaxel, cisplatin, and 5‐fluorouracil (DCF) administration. ORs are calculated with reference to patients in the cisplatin and 5‐fluorouracil (CF) group.

Figure 1 shows the results of subgroup analyses. All subgroup analyses demonstrated results consistent with the main analyses.

## 4 DISCUSSION

This nationwide database study of older patients undergoing esophagectomy after NAC found that DCF was not associated with a higher incidence of major or respiratory complications compared with CF. To the best of our knowledge, this is the largest study evaluating the perioperative outcomes of esophagectomy in older patients receiving docetaxel‐based triplet NAC.

Postoperative complications after esophagectomy would be detrimental to the long‐term prognosis, especially in older patients.^26^ Thus, preventing these complications is crucial in older patients. The present study found no significant difference in major postoperative or respiratory complications between the CF and DCF groups. The robustness of this finding was cemented by the sensitivity analyses and subgroup analyses. The other complications (i.e., respiratory failure and anastomotic leakage) and short‐term (30‐ and 90‐day) mortality were also similar between the two groups. Furthermore, 30‐day unplanned readmission did not differ between the two NAC regimens. Because early unplanned readmission can reportedly predict short‐, medium‐, and long‐term non‐cancer mortality in older patients,^27^ neoadjuvant DCF would not exert a deleterious effect on the long‐term prognosis by inducing short‐term complications even in the older patients.

DCF was associated with a higher incidence of adverse events such as febrile neutropenia compared with CF, in line with previous studies.^7^ Although advanced age has been reported to be a risk factor for febrile neutropenia,^28^ its incidence in the DCF group (18%) was comparable to findings from previous studies in younger patients.^7^ Additionally, although anorexia is one of the most common adverse events in attendant with DCF,^7^ the proportion of weight loss ≥5% during NAC did not differ between the two groups. We previously reported that weight loss ≥5% during NAC and low body mass index were independent risk factors for mortality and failure to rescue (i.e., death among patients with major complications) after esophagectomy.^19^ Furthermore, a low body mass index has been reported to be an independent factor for recurrence‐free and overall survival in older patients with esophageal cancer.^29^, ^30^ Even though DCF induced anorexia, it did not decrease body weight in older patients, compared with CF. Adverse events during NAC can lead to delayed surgery; however, the proportion of delayed surgery did not differ between the two groups. Therefore, we inferred that neoadjuvant DCF was well tolerated in older patients.

Pathological stage has also been reported to be an independent prognostic factor in older patients with esophageal cancer.^29^, ^30^ According to a prior retrospective study, DCF yielded a better pathological response compared with CF in patients aged above 75 years, comparable to that in younger patients.^10^ Moreover, a recent cohort study of esophagectomy reported that patients who achieved a pathological complete response had better overall survival, irrespective of age (<75 or ≥75 years).^31^ As DCF administration was not associated with poor perioperative outcomes (e.g., postoperative complications, short‐term mortality, and 30‐day unplanned readmission) in older patients, DCF can theoretically improve survival in older patients by virtue of the high response rates.

This study has several limitations. First, patients who were unable to undergo esophagectomy due to toxicity or disease progression during NAC were not included in the study. This is because we could not determine from the database whether the chemotherapy was for neoadjuvant or palliative purposes. However, NAC‐related severe adverse events that can render surgery infeasible are considered rare^7^, ^9^ and progression to unresectable disease is likely to occur similarly for both regimens.^7^ Thus, the exclusion of such population would not affect the current discussion on the tolerability of DCF. Second, information on the living conditions (i.e., living alone or with family) was unavailable in the database. Living alone and advanced age reportedly constitute risk factors for adverse events during NAC^28^; thus, living alone may have influenced the choice of NAC regimen for older patients. However, the instrumental variable analyses could have adjusted for such unmeasured confounders. Third, information on the dose intensity of chemotherapy was unavailable because the database contained no data on detailed dosages smaller than a vial unit. In older patients, CF and DCF regimens would be modified (e.g., dose reduction at the first cycle or pre‐planned limited cycles) depending on the status of each patient and physicians' preferences. Although factors, including comorbidities and activities of daily living, were adjusted in this analysis, these modifications may affect both short‐ and long‐term outcomes. Fourth, we did not compare neoadjuvant DCF and upfront surgery, although some hospitals prefer upfront surgery for older patients.^32^ Recently, a single‐center, retrospective study (*n* = 155) showed that (i) the incidence of postoperative complications in older patients was similar between NAC (mainly DCF) and upfront surgery, and (ii) patients with a good pathological response to NAC had a better prognosis than those with a poor response and those who underwent upfront surgery; however, these analyses were not adjusted for patient background characteristics.^33^ Because our study aimed to compare the safety between neoadjuvant CF and DCF in older patients who can tolerate NAC, we did not compare neoadjuvant DCF and upfront surgery. Finally, patients who received oxaliplatin‐based NAC (i.e., oxaliplatin and 5‐fluorouracil/leucovorin [FOLFOX] and FLOT) were not included in the study because FLOT was seldom used in Japan during the study period. However, the current study demonstrated the safety of adding docetaxel to neoadjuvant CF in older patients and a recent study reported the safety of neoadjuvant FOLFOX in older patients ineligible for CF.^34^ Therefore, we speculated that neoadjuvant FLOT (i.e., the addition of docetaxel to FOLFOX) may also be safe in older patients.

In conclusion, neoadjuvant DCF was not associated with increased postoperative complications or other perioperative adverse outcomes compared with CF. Based on our results, we do not recommend avoiding neoadjuvant DCF based on advanced age alone in patients with locally advanced esophageal cancer.

## AUTHOR CONTRIBUTIONS

**Yuki Hirano:** Conceptualization; data curation; formal analysis; investigation; methodology; project administration; visualization; writing – original draft. **Takaaki Konishi:** Data curation; methodology; validation; writing – review and editing. **Hidehiro Kaneko:** Data curation; methodology; writing – review and editing. **Satoru Matsuda:** Methodology; writing – review and editing. **Hirofumi Kawakubo:** Methodology; writing – review and editing. **Yuya Kimura:** Data curation; resources; writing – review and editing. **Hiroki Matsui:** Data curation; resources; writing – review and editing. **Kiyohide Fushimi:** Data curation; resources; writing – review and editing. **Hiroyuki Daiko:** Supervision; writing – review and editing. **Osamu Itano:** Supervision; writing – review and editing. **Hideo Yasunaga:** Conceptualization; funding acquisition; resources; supervision; writing – review and editing. **Yuko Kitagawa:** Conceptualization; supervision; writing – review and editing.

## FUNDING INFORMATION

This work was supported by grants conferred by the Ministry of Health, Labour and Welfare, Japan (23AA2003 and 24AA2006).

## CONFLICT OF INTEREST STATEMENT

Dr. Kitagawa is Editor‐in‐Chief of *Annals of Gastroenterological Surgery*. Dr. Itano and Dr. Matsuda are members of editorial board of *Annals of Gastroenterological Surgery*. Dr. Kitagawa has received lecture fees from Kyouwa Hakkou Kirin Co. Ltd., Takeda Pharmaceutical Co. Ltd., Taiho Pharmaceutical Co. Ltd., and Nippon Kayaku Co. Ltd., and research expenses, scholarship donations (grants) from Kyouwa Hakkou Kirin Co. Ltd., Takeda Pharmaceutical Co. Ltd., Taiho Pharmaceutical Co. Ltd., Nippon Kayaku Co. Ltd., Ono Pharmaceutical Co. Ltd., Bristol‐Myers Squibb K.K., and MSD K.K., which fall outside the submitted work. Dr. Konishi received grants from Pfizer Co. Ltd., Kanzawa Medical Research Foundation, and the Japan Kampo Medicines Manufacturers Association, which fall outside the submitted work. There are no other conflicts of interest to disclose.

## ETHICS STATEMENT

Approval of the research protocol by an Institutional Reviewer Board: This study was approved by the Institutional Review Board of the University of Tokyo (approval number: 3501‐[5]).

Informed Consent: The need for informed consent was waived since the data were anonymized.

Registry and the Registration No. of the study/trial: N/A.

Animal Studies: N/A.

## Supporting information


Figure S1.

Appendices S1–S2.
Tables S1–S5.

## Data Availability

Because the data in this study was extracted from a nationwide database, data use requires approval of the Ministry of Health, Labour, and Welfare, Japan.
