# Supplementary figures and images for: Perioperative outcomes of esophagectomy after doublet versus docetaxel‐based triplet neoadjuvant chemotherapy in older patients: A nationwide inpatient database study in Japan
Source: Ann Gastroenterol Surg. 2025 Feb 5;9(4):687–97. doi: 10.1002/ags3.70000 (PMC12211090; doi:10.1002/ags3.70000)

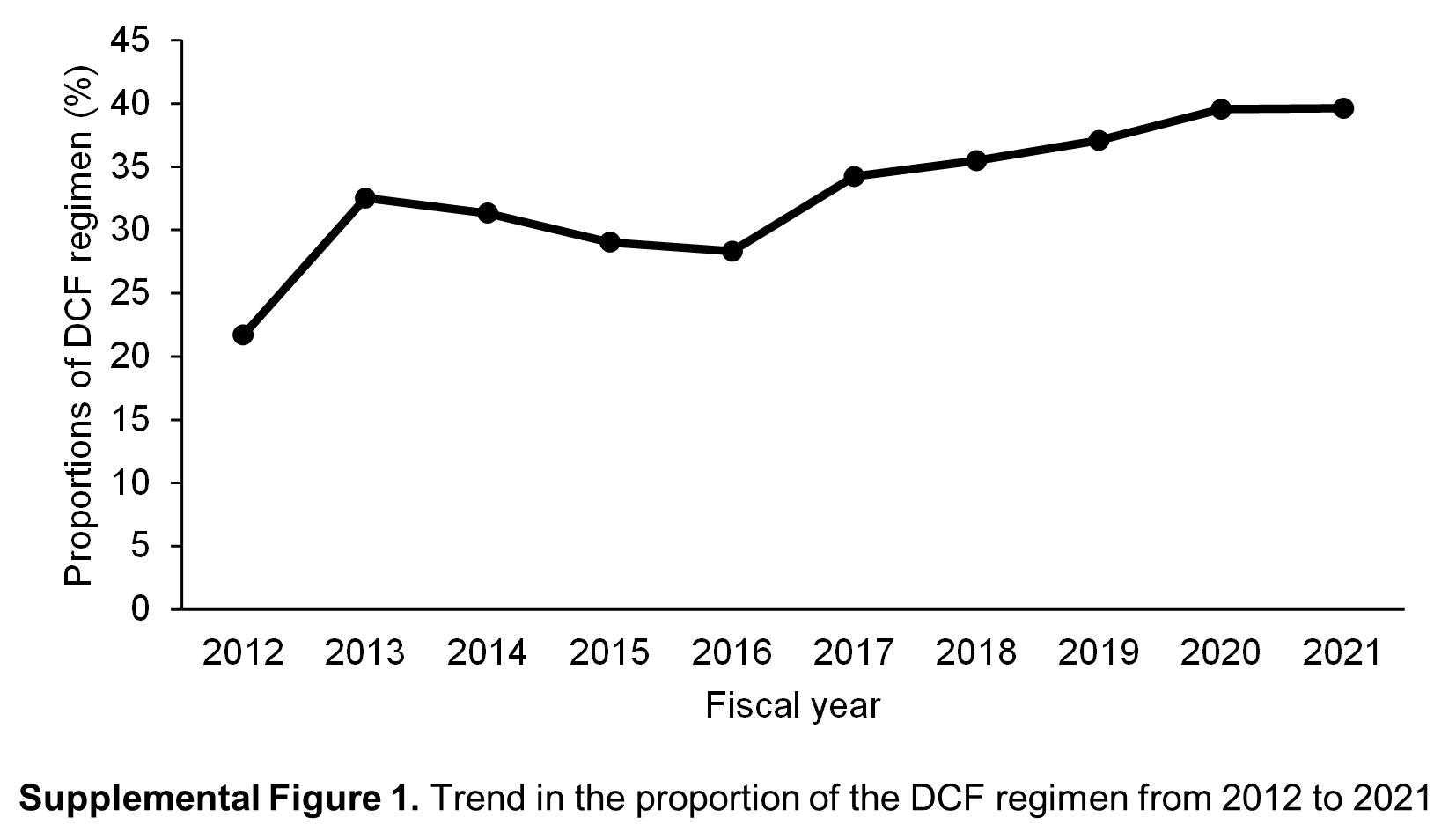

Supplement: Supplementary file 1 — Figure S1. [file AGS3-9-687-s001.tif]
