# Supplementary material for: Perioperative outcomes of esophagectomy after doublet versus docetaxel‐based triplet neoadjuvant chemotherapy in older patients: A nationwide inpatient database study in Japan
Source: Ann Gastroenterol Surg. 2025 Feb 5;9(4):687–97. doi: 10.1002/ags3.70000 (PMC12211090; doi:10.1002/ags3.70000)
Supplement: Supplementary file 2 — Appendices S1–S2. Tables S1–S5. [file AGS3-9-687-s002.docx]

**Perioperative outcomes of esophagectomy after doublet versus docetaxel-based triplet neoadjuvant chemotherapy in older patients: A nationwide inpatient database study in Japan**

Yuki Hirano, Takaaki Konishi, Hidehiro Kaneko, Satoru Matsuda, Hirofumi Kawakubo, Yuya Kimura, Hiroki Matsui, Kiyohide Fushimi, Hiroyuki Daiko, Osamu Itano, Hideo Yasunaga, and Yuko Kitagawa

**Supplemental Appendix 1.** Definitions of adverse events during neoadjuvant chemotherapy

**Supplemental Appendix 2.** Descriptions of background characteristics

**Supplemental Table 1.** ICD-10 codes and procedures used to define each postoperative complication

**Supplemental Table 2.** Adverse events during neoadjuvant chemotherapy and delayed surgery before and after overlap weighting

**Supplemental Table 3.** Patient and hospital characteristics of the propensity score-matched patients

**Supplemental Table 4.** Patient and hospital characteristics before and after overlap weighting using the redefined model (based on receipt of ≥ 2 cycles of neoadjuvant chemotherapy)

**Supplemental Table 5.** Patient and hospital characteristics before and after overlap weighting, excluding patients who underwent delayed surgery

**Supplemental Appendix 1. Definitions of adverse events during neoadjuvant chemotherapy**

We examined adverse events during neoadjuvant chemotherapy (NAC), including febrile neutropenia and weight loss. Febrile neutropenia was defined by the original Japanese code and the use of empirical broad-spectrum antibiotics from the day after commencement of the initial NAC regimen to the day before surgery. Empirical broad-spectrum antibiotics included piperacillin/tazobactam, carbapenems, fourth-generation cephalosporins, third-generation cephalosporins with anti-pseudomonal activity, quinolones plus lincomycin, quinolones plus vancomycin, or monobactams plus vancomycin, which were commonly administered to patients with febrile neutropenic (including those with allergy or intolerance to beta-lactams) during the study period.^1^ Relative percent weight loss during NAC was calculated using the weight at admission for the initial NAC (pre-NAC weight) and the weight at admission for surgery (pre-surgery weight), i.e. weight change = (pre-surgery weight – pre-NAC weight)/pre-NAC weight × 100.^2^

**Supplemental Appendix 2. Descriptions of background characteristics**

We examined patient-related background factors prior to NAC, including sex, age, body mass index, activities of daily living (Barthel index < 95, inability to walk independently [> 45 m]),^3^ pre-NAC tube feeding, smoking index, Charlson comorbidity index, hypertension, diabetes mellitus, chronic obstructive pulmonary disease, liver disease, clinical T factor, clinical N factor, and clinical M factor. Data on age, body mass index, and activities of daily living at admission for initial NAC were included in the analysis. The body mass index was categorized into five groups based on the criteria for Asia-Pacific populations formulated by the World Health Organization, in accordance with our previous study: < 16.0 kg/m^2^ (severe underweight), 16.0–18.4 kg/m^2^ (mild-to-moderate underweight), 18.5–22.9 kg/m^2^ (normal), 23.0–27.4 kg/m^2^ (overweight), and ≥ 27.5 kg/m^2^ (obese).^4, 5^ Pre-NAC tube feeding was defined as enteral nutrition via jejunostomy, gastrostomy, nasojejunal tube, or nasogastric tube initiated before the initiation of NAC.^6^ The smoking index was categorized into five groups: 0–5, 6–20, 21–40, ≥ 41 pack-years, and missing. Comorbidities were scored using the Charlson comorbidity index using the protocol established by Quan et al. from the International Classification of Diseases, Tenth Revision (ICD-10) codes^7^ and classified into three groups: 2, 3–4, and ≥ 5.^8, 9^

Additionally, we examined hospital-related factors (hospital type, hospital volume, hospitals’ early extubation proportion, hospitals’ minimally invasive oesophagectomy proportion, and fiscal year). The type of hospital was teaching or non-teaching. Hospital volume was defined as the number of all esophagectomies performed annually at each hospital. The proportion of patients who underwent early extubation at each hospital during the study period was examined; early extubation was defined as extubation on the day of surgery without the need for mechanical ventilation on the day after surgery.^10, 11^ The proportion of patients who received minimally invasive esophagectomy at each hospital between April 2014 and March 2022 was examined. Minimally invasive esophagectomy was defined as esophagectomy using the minimally invasive thoracic approach (thoracoscopic, mediastinoscopy-assisted, or robot-assisted).^4, 11^ Information on the thoracic approach was not recorded before April 2014. Hospital volume, hospitals’ early extubation proportion, and hospitals’ minimally invasive esophagectomy proportion were categorized into four groups with approximately equal numbers of patients in each group.

REFERENCES

1. Nomura H, Hatogai K, Maki Y, et al. Risk factors for febrile neutropenia in neoadjuvant docetaxel, cisplatin, and 5-fluorouracil chemotherapy for esophageal cancer. *Support Care Cancer.* 2020;28:1849–1854.

2. Hirano Y, Konishi T, Kaneko H, et al. Weight loss during neoadjuvant therapy and short-term outcomes after esophagectomy: A retrospective cohort study. *Int J Surg.* 2023;109:805–812.

3. Mahoney FI, Barthel DW. Functional evaluation: The Barthel index. *Md State Med J.* 1965;14:61–65.

4. Hirano Y, Kaneko H, Konishi T, et al. Impact of body mass index on major complications, multiple complications, in-hospital mortality, and failure to rescue after esophagectomy for esophageal cancer: A nationwide inpatient database study in Japan. *Ann Surg.* 2023;277:e785–e792.

5. WHO Expert Consultation. Appropriate body-mass index for Asian populations and its implications for policy and intervention strategies. *Lancet*. 2004;363:157-163.

6. Hirano Y, Konishi T, Kaneko H, et al. Weight loss during neoadjuvant therapy and short-term outcomes after esophagectomy: A retrospective cohort study. *Int J Surg.* 2023;109:805–812.

7. Quan H, Sundararajan V, Halfon P, et al. Coding algorithms for defining comorbidities in ICD-9-CM and ICD-10 administrative data. *Med Care*. 2005;43:1130-1139.

8. Quan H, Li B, Couris CM, Graham P, et al. Updating and validating the Charlson comorbidity index and score for risk adjustment in hospital discharge abstracts using data from 6 countries. *Am J Epidemiol.* 2011;173:676-682.

9. Bannay A, Chaignot C, Blotière PO, et al. The best use of the Charlson comorbidity index with an electronic health care database to predict mortality. *Med Care*. 2016;54:188-194.

10. Hirano Y, Konishi T, Kaneko H, et al. Proportion of early extubation and short-term outcomes after esophagectomy: A retrospective cohort study. *Int J Surg.* 2023;109:3097–3106.

11. Hirano Y, Konishi T, Kaneko H, et al. Antimicrobial prophylaxis with ampicillin-sulbactam compared with cefazolin for esophagectomy: Nationwide inpatient database study in Japan. *Ann Surg.* 2024;279:640–647.

**Supplemental Table 1.** **ICD-10 codes and procedures used to define each postoperative complication**

|  | **ICD-10 codes** | **Procedures (searched in the original Japanese codes)** |
| --- | --- | --- |
| *Major complications* |  |  |
| Respiratory complications | J12–18, J80, J96, J690, J691, J958, J959 | Mechanical ventilation use lasting > 2 days following surgery |
| Anastomotic leakage | T813 | Long-term drainage tube placement (defined as insertion of a drainage tube for ≥ 3 weeks after surgery) or procedures for the management of anastomotic leakage |
| Pneumothorax | J93 | Procedures for the management of pneumothorax |
| Chylothorax | I898, S278, T812 | Procedures for the management of chylothorax |
| Empyema | J860, J869 | Procedures for empyema |
| Peritonitis | K65 | Procedures for peritonitis |
| Ileus/bowel obstruction/symptomatic hernia (hiatal or diaphragmatic) | K560, K562, K565–567, K913, K440, K441, K449 | Procedures for the management of ileus/bowel obstruction or hiatal/diaphragmatic hernia |
| Pulmonary embolism | I26 | None |
| Acute coronary syndrome | I21–25 | None |
| Heart failure | I50 | None |
| Stroke | I60–66 | None |
| Acute kidney injury | N17 | None |
| Sepsis | A021, A227, A241, A267, A282, A327, A394, A40, A41, A548, B007, B349, B377, P36 | None |

ICD-10, International Classification of Diseases, Tenth Revision

**Supplemental Table 2.** **Adverse events during neoadjuvant chemotherapy and delayed surgery before and after overlap weighting**

| **Outcome** | **Before overlap weighting** | | ***P* value** | **After overlap weighting^†^** | | | ***P* value** |
| --- | --- | --- | --- | --- | --- | --- | --- |
|  | **CF** | **DCF** |  | **CF** | **DCF** | **Odds ratio or coefficient (95% CI)** |  |
|  | **(*n* = 3457)** | **(*n* = 1772)** |  | **(*n* = 2609)** | **(*n* = 2609)** |  |  |
| **Adverse events** |  |  |  |  |  |  |  |
| Febrile neutropenia | 31 (0.9) | 311 (18) | <0.001 | (1.1) | (17) | 18.3 (12.0–27.7) | <0.001 |
| Median weight loss, % | 2.2 (-0.3 to 5.4) | 1.6 (-1.8 to 5.3) | <0.001 | 2.3 (-0.2 to 5.4) | 1.6 (-1.8 to 5.2) | -1.1 (-0.6 to -1.5) | <0.001 |
| Weight loss ≥ 5% | 905 (26) | 453 (26) | 0.63 | (26) | (25) | 0.93 (0.81–1.07) | 0.32 |
| **Delayed surgery** | 280 (8.1) | 154 (8.7) | 0.46 | (8.5) | (9.6) | 1.13 (0.91–1.42) | 0.27 |

Data are presented as *n* (%) or median (interquartile range).

Odds ratios or coefficients are calculated with reference to patients in the CF group.

CF, cisplatin, 5-fluorouracil; DCF, docetaxel, cisplatin, 5-fluorouracil; CI, confidence interval

^†^After overlap weighting, one individual no longer represents one data entity; thus, the raw counts are not reported after overlap weighting.

**Supplemental Table 3.** **Patient and hospital characteristics of the propensity score-matched patients**

| **Variable** | **Propensity score matching** | |  |
| --- | --- | --- | --- |
|  | **CF** | **DCF** | **ASD** |
|  | **(*n* = 1425)** | **(*n* = 1425)** |  |
| **Sex,** male | 1211 (85) | 1218 (85) | 1.4 |
| **Age,** years |  |  |  |
| 70–71 | 435 (31) | 432 (30) | 0.5 |
| 72–73 | 390 (27) | 389 (27) | 0.2 |
| 74–75 | 287 (20) | 301 (21) | 2.4 |
| 76–77 | 188 (13) | 192 (13) | 0.8 |
| 78–79 | 125 (8.8) | 111 (7.8) | 3.6 |
| **Body mass index,** kg/m^2^ |  |  |  |
| < 16.0 | 47 (3.3) | 37 (2.6) | 4.2 |
| 16.0–18.4 | 184 (13) | 175 (12) | 1.9 |
| 18.5–22.9 | 788 (55) | 760 (53) | 3.9 |
| 23.0–27.4 | 366 (26) | 406 (28) | 6.3 |
| ≥ 27.5 | 40 (2.8) | 47 (3.3) | 2.9 |
| **Activities of daily living** |  |  |  |
| Barthel index < 95 | 50 (3.5) | 38 (2.7) | 4.9 |
| Inability to walk independently (> 45 m) | 38 (2.7) | 28 (2.0) | 4.7 |
| **Pre-NAC tube feeding** | 76 (5.3) | 80 (5.6) | 1.2 |
| **Smoking index**, pack-years |  |  |  |
| 0–5 | 416 (29) | 412 (29) | 0.6 |
| 6–20 | 149 (10) | 158 (11) | 2.0 |
| 21–40 | 293 (21) | 296 (21) | 0.5 |
| ≥ 41 | 423 (30) | 411 (29) | 1.9 |
| Missing | 144 (10) | 148 (10) | 0.9 |
| **Comorbidities** |  |  |  |
| Charlson comorbidity index |  |  |  |
| 2 | 1139 (80) | 1141 (80) | 0.4 |
| 3–4 | 183 (13) | 183 (13) | 0.0 |
| ≥ 5 | 103 (7.2) | 101 (7.1) | 0.5 |
| Hypertension | 409 (29) | 408 (29) | 0.2 |
| Diabetes mellitus | 175 (12) | 187 (13) | 2.5 |
| Chronic obstructive pulmonary disease | 31 (2.2) | 31 (2.2) | 0.0 |
| Liver disease | 46 (3.2) | 47 (3.3) | 0.4 |
| **Clinical T factor** |  |  |  |
| T1 | 149 (10) | 149 (10) | 0.0 |
| T2 | 256 (18) | 239 (17) | 3.1 |
| T3 | 854 (60) | 870 (61) | 2.3 |
| T4 | 79 (5.5) | 76 (5.3) | 0.9 |
| TX/missing | 87 (6.1) | 91 (6.4) | 1.2 |
| **Clinical N factor** |  |  |  |
| N0 | 308 (22) | 328 (23) | 3.4 |
| N1 | 584 (41) | 549 (39) | 5.0 |
| N2–3 | 448 (31) | 462 (32) | 2.1 |
| NX/missing | 85 (6.0) | 86 (6.0) | 0.3 |
| **Clinical M factor** |  |  |  |
| M0 | 1249 (88) | 1258 (88) | 1.9 |
| M1 | 83 (5.8) | 72 (5.1) | 3.4 |
| MX/missing | 93 (6.5) | 95 (6.7) | 0.6 |
| **Teaching hospital** | 474 (33) | 472 (33) | 0.3 |
| **Hospital volume**, cases/year |  |  |  |
| 5.0–11.0 | 229 (16) | 217 (15) | 2.3 |
| 11.1–19.4 | 310 (22) | 300 (21) | 1.7 |
| 19.5–31.5 | 318 (22) | 339 (24) | 3.5 |
| 31.6–73.0 | 279 (20) | 301 (21) | 3.8 |
| ≥ 73.1 | 289 (20) | 268 (19) | 3.7 |
| **Hospitals’ early extubation proportion** |  |  |  |
| 0–9% | 334 (23) | 350 (25) | 2.6 |
| 10–20% | 286 (20) | 276 (19) | 1.8 |
| 21–72% | 290 (20) | 284 (20) | 1.0 |
| 73–94% | 269 (19) | 261 (18) | 1.4 |
| ≥ 95% | 246 (17) | 254 (18) | 1.5 |
| **Hospitals’ MIE proportion** |  |  |  |
| 0–50% | 294 (21) | 318 (22) | 4.1 |
| 51–63% | 311 (22) | 295 (21) | 2.7 |
| 64–74% | 309 (22) | 307 (22) | 0.3 |
| 75–86% | 302 (21) | 298 (21) | 0.7 |
| ≥ 87% | 209 (15) | 207 (15) | 0.4 |
| **Fiscal year** |  |  |  |
| 2012–2013 | 155 (11) | 147 (10) | 1.8 |
| 2014–2015 | 229 (16) | 244 (17) | 2.8 |
| 2016–2017 | 279 (20) | 292 (20) | 2.3 |
| 2018–2019 | 354 (25) | 346 (24) | 1.3 |
| 2020–2021 | 408 (29) | 396 (28) | 1.9 |

Data are presented as *n* (%).

CF, cisplatin, 5-fluorouracil; DCF, docetaxel, cisplatin, 5-fluorouracil; ASD, absolute standardized difference; NAC, neoadjuvant chemotherapy; MIE, minimally invasive esophagectomy

**Supplemental Table 4. Patient and hospital characteristics before and after overlap weighting using the redefined model (based on receipt of ≥ 2 cycles of neoadjuvant chemotherapy)**

| **Variable** | **Before overlap weighting** | |  | **After overlap weighting^†^** | |  |
| --- | --- | --- | --- | --- | --- | --- |
|  | **CF** | **DCF** | **ASD** | **CF** | **DCF** | **ASD** |
|  | **(*n* = 2679)** | **(*n* = 1502)** |  | **(*n* = 2086)** | **(*n* = 2086)** |  |
| **Sex,** male | 2244 (84) | 1285 (86) | 5.0 | (85) | (85) | 0.0 |
| **Age,** years |  |  |  |  |  |  |
| 70–71 | 720 (27) | 500 (33) | 14 | (30) | (30) | 0.0 |
| 72–73 | 640 (24) | 428 (28) | 10 | (27) | (27) | 0.0 |
| 74–75 | 607 (23) | 300 (20) | 6.6 | (21) | (21) | 0.0 |
| 76–77 | 446 (17) | 182 (12) | 13 | (14) | (14) | 0.0 |
| 78–79 | 266 (9.9) | 92 (6.1) | 14 | (7.4) | (7.4) | 0.0 |
| **Body mass index,** kg/m^2^ |  |  |  |  |  |  |
| < 16.0 | 59 (2.2) | 49 (3.3) | 6.5 | (2.8) | (2.8) | 0.0 |
| 16.0–18.4 | 305 (11) | 191 (13) | 4.1 | (12) | (12) | 0.0 |
| 18.5–22.9 | 1390 (52) | 809 (54) | 4.0 | (54) | (54) | 0.0 |
| 23.0–27.4 | 835 (31) | 408 (27) | 8.8 | (28) | (28) | 0.0 |
| ≥ 27.5 | 90 (3.4) | 45 (3.0) | 2.1 | (3.3) | (3.3) | 0.0 |
| **Activities of daily living** |  |  |  |  |  |  |
| Barthel index < 95 | 61 (2.3) | 100 (6.7) | 21 | (3.3) | (3.3) | 0.0 |
| Inability to walk independently (> 45 m) | 43 (1.6) | 81 (5.4) | 21 | (2.5) | (2.5) | 0.0 |
| **Pre-NAC tube feeding** | 99 (3.7) | 114 (7.6) | 17 | (5.3) | (5.3) | 0.0 |
| **Smoking index**, pack-years |  |  |  |  |  |  |
| 0–5 | 769 (29) | 443 (29) | 1.7 | (29) | (29) | 0.0 |
| 6–20 | 295 (11) | 167 (11) | 0.3 | (11) | (11) | 0.0 |
| 21–40 | 571 (21) | 304 (20) | 2.6 | (21) | (21) | 0.0 |
| ≥ 41 | 755 (28) | 456 (30) | 4.8 | (30) | (30) | 0.0 |
| Missing | 289 (11) | 132 (8.8) | 6.7 | (9.7) | (9.7) | 0.0 |
| **Comorbidities** |  |  |  |  |  |  |
| Charlson comorbidity index |  |  |  |  |  |  |
| 2 | 2132 (80) | 1188 (79) | 1.2 | (80) | (80) | 0.0 |
| 3–4 | 386 (14) | 204 (14) | 2.4 | (13) | (13) | 0.0 |
| ≥ 5 | 161 (6.0) | 110 (7.3) | 5.3 | (6.9) | (6.9) | 0.0 |
| Hypertension | 734 (27) | 425 (28) | 2.0 | (28) | (28) | 0.0 |
| Diabetes mellitus | 399 (15) | 181 (12) | 8.3 | (13) | (13) | 0.0 |
| Chronic obstructive pulmonary disease | 93 (3.5) | 36 (2.4) | 6.4 | (2.6) | (2.6) | 0.0 |
| Liver disease | 123 (4.6) | 44 (2.9) | 8.7 | (3.5) | (3.5) | 0.0 |
| **Clinical T factor** |  |  |  |  |  |  |
| T1 | 417 (16) | 134 (8.9) | 20 | (12) | (12) | 0.0 |
| T2 | 613 (23) | 217 (14) | 22 | (18) | (18) | 0.0 |
| T3 | 1420 (53) | 921 (61) | 17 | (60) | (60) | 0.0 |
| T4 | 79 (2.9) | 142 (9.5) | 27 | (5.4) | (5.4) | 0.0 |
| TX/missing | 150 (5.6) | 88 (5.9) | 1.1 | (5.9) | (5.9) | 0.0 |
| **Clinical N factor** |  |  |  |  |  |  |
| N0 | 743 (28) | 331 (22) | 13 | (24) | (24) | 0.0 |
| N1 | 1118 (42) | 564 (38) | 8.6 | (39) | (39) | 0.0 |
| N2–3 | 665 (25) | 529 (35) | 23 | (31) | (31) | 0.0 |
| NX/missing | 153 (5.7) | 78 (5.2) | 2.3 | (5.6) | (5.6) | 0.0 |
| **Clinical M factor** |  |  |  |  |  |  |
| M0 | 2444 (91) | 1288 (86) | 17 | (89) | (89) | 0.0 |
| M1 | 84 (3.1) | 127 (8.5) | 23 | (5.2) | (5.2) | 0.0 |
| MX/missing | 151 (5.6) | 87 (5.8) | 0.7 | (6.1) | (6.1) | 0.0 |
| **Teaching hospital** | 950 (35) | 443 (29) | 13 | (33) | (33) | 0.0 |
| **Hospital volume**, cases/year |  |  |  |  |  |  |
| 5.0–11.0 | 625 (23) | 182 (12) | 30 | (16) | (16) | 0.0 |
| 11.1–19.4 | 571 (21) | 273 (18) | 7.9 | (21) | (21) | 0.0 |
| 19.5–31.5 | 527 (20) | 321 (21) | 4.2 | (23) | (23) | 0.0 |
| 31.6–73.0 | 528 (20) | 299 (20) | 0.5 | (20) | (20) | 0.0 |
| ≥ 73.1 | 428 (16) | 427 (28) | 30 | (20) | (20) | 0.0 |
| **Hospitals’ early extubation proportion** |  |  |  |  |  |  |
| 0–9% | 480 (18) | 346 (23) | 13 | (22) | (22) | 0.0 |
| 10–20% | 422 (16) | 412 (27) | 29 | (19) | (19) | 0.0 |
| 21–72% | 563 (21) | 257 (17) | 10 | (20) | (20) | 0.0 |
| 73–94% | 557 (21) | 237 (16) | 13 | (19) | (19) | 0.0 |
| ≥ 95% | 657 (25) | 250 (17) | 20 | (19) | (19) | 0.0 |
| **Hospitals’ MIE proportion** |  |  |  |  |  |  |
| 0–50% | 503 (19) | 293 (20) | 1.9 | (21) | (21) | 0.0 |
| 51–63% | 499 (19) | 391 (26) | 18 | (22) | (22) | 0.0 |
| 64–74% | 541 (20) | 307 (20) | 0.6 | (21) | (21) | 0.0 |
| 75–86% | 479 (18) | 330 (22) | 10 | (20) | (20) | 0.0 |
| ≥ 87% | 649 (24) | 180 (12) | 32 | (16) | (16) | 0.0 |
| **Fiscal year** |  |  |  |  |  |  |
| 2012–2013 | 332 (12) | 143 (9.5) | 9.2 | (10) | (10) | 0.0 |
| 2014–2015 | 495 (18) | 233 (16) | 7.9 | (16) | (16) | 0.0 |
| 2016–2017 | 588 (22) | 300 (20) | 4.9 | (20) | (20) | 0.0 |
| 2018–2019 | 625 (23) | 387 (26) | 5.7 | (25) | (25) | 0.0 |
| 2020–2021 | 639 (24) | 439 (29) | 12 | (28) | (28) | 0.0 |

Data are presented as *n* (%).

CF, cisplatin, 5-fluorouracil; DCF, docetaxel, cisplatin, 5-fluorouracil; ASD, absolute standardized difference; NAC, neoadjuvant chemotherapy; MIE, minimally invasive esophagectomy

^†^After overlap weighting, one individual no longer represents one data entity and thus raw counts are not reported after overlap weighting.

**Supplemental Table 5. Patient and hospital characteristics before and after overlap weighting, excluding patients who underwent delayed surgery**

| **Variable** | **Before overlap weighting** | |  | **After overlap weighting^†^** | |  |
| --- | --- | --- | --- | --- | --- | --- |
|  | **CF** | **DCF** | **ASD** | **CF** | **DCF** | **ASD** |
|  | **(*n* = 3177)** | **(*n* = 1618)** |  | **(*n* = 2393)** | **(*n* = 2393)** |  |
| **Sex,** male | 2640 (83) | 1385 (86) | 6.9 | (85) | (85) | 0.0 |
| **Age,** years |  |  |  |  |  |  |
| 70–71 | 843 (27) | 537 (33) | 15 | (30) | (30) | 0.0 |
| 72–73 | 784 (25) | 449 (28) | 7.0 | (27) | (27) | 0.0 |
| 74–75 | 677 (21) | 323 (20) | 3.3 | (21) | (21) | 0.0 |
| 76–77 | 552 (17) | 203 (13) | 14 | (14) | (14) | 0.0 |
| 78–79 | 321 (10) | 106 (6.6) | 13 | (7.8) | (7.8) | 0.0 |
| **Body mass index,** kg/m^2^ |  |  |  |  |  |  |
| < 16.0 | 73 (2.3) | 55 (3.4) | 6.6 | (3.0) | (3.0) | 0.0 |
| 16.0–18.4 | 366 (12) | 211 (13) | 4.6 | (12) | (12) | 0.0 |
| 18.5–22.9 | 1652 (52) | 868 (54) | 3.3 | (54) | (54) | 0.0 |
| 23.0–27.4 | 979 (31) | 436 (27) | 8.5 | (28) | (28) | 0.0 |
| ≥ 27.5 | 107 (3.4) | 48 (3.0) | 2.3 | (3.1) | (3.1) | 0.0 |
| **Activities of daily living** |  |  |  |  |  |  |
| Barthel index < 95 | 121 (3.8) | 117 (7.2) | 15 | (3.5) | (3.5) | 0.0 |
| Inability to walk independently (> 45 m) | 74 (2.3) | 115 (7.1) | 23 | (2.6) | (2.6) | 0.0 |
| **Pre-NAC tube feeding** | 49 (1.5) | 94 (5.8) | 23 | (5.3) | (5.3) | 0.0 |
| **Smoking index**, pack-years |  |  |  |  |  |  |
| 0–5 | 947 (30) | 487 (30) | 0.6 | (29) | (29) | 0.0 |
| 6–20 | 339 (11) | 177 (11) | 0.9 | (11) | (11) | 0.0 |
| 21–40 | 668 (21) | 339 (21) | 0.2 | (21) | (21) | 0.0 |
| ≥ 41 | 862 (27) | 464 (29) | 3.4 | (28) | (28) | 0.0 |
| Missing | 361 (11) | 151 (9.3) | 6.7 | (10) | (10) | 0.0 |
| **Comorbidities** |  |  |  |  |  |  |
| Charlson comorbidity index |  |  |  |  |  |  |
| 2 | 2539 (80) | 1303 (81) | 1.5 | (81) | (81) | 0.0 |
| 3–4 | 456 (14) | 203 (13) | 5.3 | (12) | (12) | 0.0 |
| ≥ 5 | 182 (5.7) | 112 (6.9) | 4.9 | (6.6) | (6.6) | 0.0 |
| Hypertension | 867 (27) | 462 (29) | 2.8 | (29) | (29) | 0.0 |
| Diabetes mellitus | 461 (15) | 199 (12) | 6.5 | (13) | (13) | 0.0 |
| Chronic obstructive pulmonary disease | 96 (3.0) | 30 (1.9) | 7.6 | (2.0) | (2.0) | 0.0 |
| Liver disease | 153 (4.8) | 47 (2.9) | 9.9 | (3.5) | (3.5) | 0.0 |
| **Clinical T factor** |  |  |  |  |  |  |
| T1 | 483 (15) | 140 (8.7) | 20 | (11) | (11) | 0.0 |
| T2 | 706 (22) | 234 (14) | 20 | (17) | (17) | 0.0 |
| T3 | 1707 (54) | 1010 (62) | 18 | (60) | (60) | 0.0 |
| T4 | 101 (3.2) | 138 (8.5) | 23 | (5.4) | (5.4) | 0.0 |
| TX/missing | 180 (5.7) | 96 (5.9) | 1.1 | (6.2) | (6.2) | 0.0 |
| **Clinical N factor** |  |  |  |  |  |  |
| N0 | 887 (28) | 339 (21) | 16 | (24) | (24) | 0.0 |
| N1 | 1311 (41) | 613 (38) | 6.9 | (39) | (39) | 0.0 |
| N2–3 | 792 (25) | 577 (36) | 24 | (32) | (32) | 0.0 |
| NX/missing | 187 (5.9) | 89 (5.5) | 1.7 | (6.0) | (6.0) | 0.0 |
| **Clinical M factor** |  |  |  |  |  |  |
| M0 | 2895 (91) | 1382 (85) | 18 | (88) | (88) | 0.0 |
| M1 | 99 (3.1) | 138 (8.5) | 23 | (5.3) | (5.3) | 0.0 |
| MX/missing | 183 (5.8) | 98 (6.1) | 1.3 | (6.4) | (6.4) | 0.0 |
| **Teaching hospital** | 1113 (35) | 436 (27) | 18 | (31) | (31) | 0.0 |
| **Hospital volume**, cases/year |  |  |  |  |  |  |
| 5.0–11.0 | 754 (24) | 192 (12) | 31 | (16) | (16) | 0.0 |
| 11.1–19.4 | 660 (21) | 294 (18) | 6.6 | (21) | (21) | 0.0 |
| 19.5–31.5 | 626 (20) | 323 (20) | 0.6 | (22) | (22) | 0.0 |
| 31.6–73.0 | 654 (21) | 327 (20) | 0.9 | (21) | (21) | 0.0 |
| ≥ 73.1 | 483 (15) | 482 (30) | 35 | (20) | (20) | 0.0 |
| **Hospitals’ early extubation proportion** |  |  |  |  |  |  |
| 0–9% | 582 (18) | 371 (23) | 11 | (23) | (23) | 0.0 |
| 10–20% | 496 (16) | 458 (28) | 31 | (19) | (19) | 0.0 |
| 21–72% | 683 (21) | 284 (18) | 10 | (21) | (21) | 0.0 |
| 73–94% | 671 (21) | 259 (16) | 13 | (20) | (20) | 0.0 |
| ≥ 95% | 745 (23) | 246 (15) | 21 | (17) | (17) | 0.0 |
| **Hospitals’ MIE proportion** |  |  |  |  |  |  |
| 0–50% | 606 (19) | 312 (19) | 0.5 | (21) | (21) | 0.0 |
| 51–63% | 560 (18) | 435 (27) | 22 | (22) | (22) | 0.0 |
| 64–74% | 633 (20) | 324 (20) | 0.3 | (21) | (21) | 0.0 |
| 75–86% | 569 (18) | 366 (23) | 12 | (21) | (21) | 0.0 |
| ≥ 87% | 802 (25) | 179 (11) | 37 | (15) | (15) | 0.0 |
| **Fiscal year** |  |  |  |  |  |  |
| 2012–2013 | 420 (13) | 159 (9.8) | 11 | (11) | (11) | 0.0 |
| 2014–2015 | 599 (19) | 259 (16) | 7.5 | (17) | (17) | 0.0 |
| 2016–2017 | 703 (22) | 313 (19) | 6.9 | (20) | (20) | 0.0 |
| 2018–2019 | 718 (23) | 399 (25) | 4.9 | (24) | (24) | 0.0 |
| 2020–2021 | 737 (23) | 488 (30) | 16 | (28) | (28) | 0.0 |

Data are presented as *n* (%).

CF, cisplatin, 5-fluorouracil; DCF, docetaxel, cisplatin, 5-fluorouracil; ASD, absolute standardized difference; NAC, neoadjuvant chemotherapy; MIE, minimally invasive esophagectomy

^†^After overlap weighting, one individual no longer represents one data entity and thus raw counts are not reported after overlap weighting.
